# Ichthyoses—A Clinical and Pathological Spectrum from Heterogeneous Cornification Disorders to Inflammation

**DOI:** 10.3390/dermatopathology8020017

**Published:** 2021-05-07

**Authors:** Dieter Metze, Heiko Traupe, Kira Süßmuth

**Affiliations:** Klinik für Hautkrankheiten, Universitätsklinik Münster, 48149 Münster, Germany; traupeh@ukmuenster.de (H.T.); Kira.Suessmuth@ukmuenster.de (K.S.)

**Keywords:** ichthyosis, hereditary keratinization disorders, dermatopathology, pattern analysis, immunohistochemistry

## Abstract

Ichthyoses are inborn keratinization disorders affecting the skin only (non-syndromic) or are associated with diseases of internal organs (syndromic). In newborns, they can be life-threatening. The identification of the gene defects resulted in reclassification and a better understanding of the pathophysiology. Histopathologic patterns include orthohyperkeratosis with a reduced or well-developed stratum granulosum, hyperkeratosis with ortho- and parakeratosis with preserved or prominent stratum granulosum, and epidermolytic ichthyosis. Another pattern features “perinuclear vacuoles and binucleated keratinocytes”, which is associated with keratin mutations. Some ichthyoses are histologically defined by psoriasis-like features, and distinct subtypes show follicular hyperkeratosis. In addition to histological and immunohistochemical methods, these patterns allow a better histopathologic diagnosis.

## 1. Target Readership

The article was written for dermatologists and pathologists interested in genodermatoses and dermatohistology, especially the diagnosis of ichthyoses. It is supposed to help to diagnose different types of ichthyoses when genetic analyses are not available or before genetic testing.

## 2. Introduction

Ichthyoses are hereditary keratinization disorders defined by universal scaling occurring over the entire body. Some forms manifest at birth (“congenital” forms), others during the first year of life (“vulgar” forms) (Table 1) [1,2].

An accurate and rapid diagnosis of a hereditary keratinization disorder is important to identify associated diseases of internal organs in syndromic forms (Table 1), to initiate genetic counseling, and to start potential therapies [1,2].

The histopathology of ichthyoses is mentioned in publications and book chapters [3] but has not been systematically studied and is often considered “nonspecific.” Traditionally, therefore, the clinical picture, family history, and occasionally laboratory chemistry and electron microscopic studies have been crucial for diagnosis. Only the identification of the genetic causes has led to an understanding of the molecular mechanisms, as well as to the reclassification of these genodermatoses (Table 1) [4,5]. Assistance is provided by special networks (www.netzwerk-ichthyose.de (accessed on 3 May 2021)) and patient support-groups (https://www.ichthyose.de/ (accessed on 3 May 2021)).

In parallel, the pathological changes of the skin biopsies were characterized in more detail. Certain histological patterns could be defined. They include the following criteria: hyperkeratosis with/without parakeratosis, expression/absence of the stratum granulosum, atrophy/hyperplasia (acanthosis) of the epidermis, vacuolization/eosinophilic granules in keratinocytes, or hyperkeratosis and a degree of development of hair follicles. Complementary histo- and immunohistochemical methods allow for, in part, a precise diagnosis, but at least a limitation of differential diagnoses, which can then be further clarified by targeted mutation analyses [6,7].

In the following, the dermatopathological diagnosis is presented on the basis of some frequent, but also rare syndromic and life-threatening ichthyoses, which can be performed quickly, easily, and economically on sample biopsies of the skin. Hereditary keratinization disorders are also discussed. They often show a highly inflammatory, psoriasis-like picture and are therefore often misdiagnosed (Table 2) [6,8].

## 3. Ichthyosis Vulgaris

**Figure 1:**
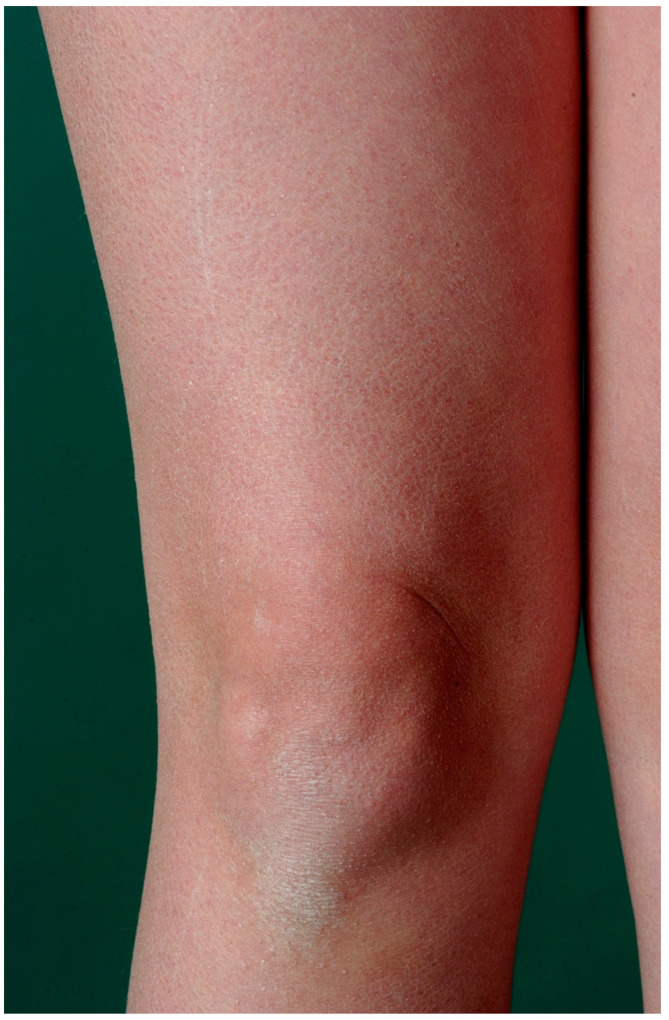
Ichthyosis vulgaris. Fine grey scaling on the extremities.

The autosomal semidominant inherited ichthyosis vulgaris is the most frequent ichthyosis (prevalence from 1:100 to 1:250) [9]. It usually develops in the course of the first year of life and manifests with dry skin or light gray fine scales (Figure 1) as well as palmoplantar hyperlinearity. The disorder is caused by loss-of-function mutations in the *filaggrin* gene (Table 3). Filaggrin is expressed in the keratohyalin granules and crosslinks the keratin filaments in the horny layer. A deficiency of filaggrin predisposes to atopic dermatitis and/or allergic rhinoconjunctivitis [8,10]. Interestingly, *filaggrin* mutations can also be observed in X-linked recessive ichthyosis underlying steroid sulfatase deficiency [10].

### 3.1. Histology

**Figure 2:**
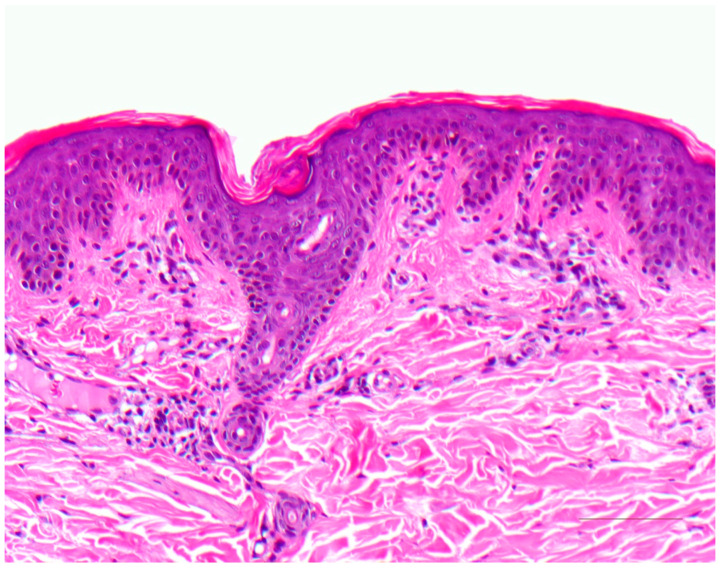
Ichthyosis vulgaris. Note the absent stratum granulosum and mild compact orthohyperkeratosis. Marked hyperkeratosis of the opening of the acrosyringium. Inflammatory infiltrates are almost absent. HE stain, bar = 100 µm.

A characteristic feature of ichthyosis vulgaris is a markedly reduced, often completely absent stratum granulosum. The stratum corneum exhibits mild compact orthohyperkeratosis (Figure 2). Frequently, there is also hyperkeratosis of hair follicles and acrosyringia. The epidermis may be slightly widened (acanthotic) but also atrophic. Isolated mild perivascular lymphocytic infiltrates can be found in the dermis. Associated signs of spongiform dermatitis may be encountered in the setting of atopy.

Immunohistochemically, a deficiency of filaggrin can be quantified, which correlates with the number of mutations (one or two mutations in the *filaggrin* gene) and thus, the severity of ichthyosis. Ultrastructurally, there is a defect in the keratohyalin granules, which appear diminished and crumbly.

### 3.2. Differential Diagnoses

A thinned or absent stratum granulosum with mild orthohyperkeratosis is also observed in patients with atopy and other very rare ichthyoses, such as Conradi–Hünermann–Happle syndrome [6]. It can also be found in acquired ichthyosis-like skin conditions (“acquired ichthyoses”). Causes are malignancies (lymphomas), renal insufficiency, Crohn′s disease, autoimmune diseases (collagenoses), GvHD, infections (HIV, leprosy), endocrinopathies (hypothyroidism), sarcoidosis, malnutrition (vitamin A) or drugs (lipid-lowering drugs, psychotropic drugs) [11].

## 4. Autosomal Recessive Congenital Ichthyosis

Autosomal recessive congenital ichthyosis (ARCI) represents a genetically heterogeneous group of non-syndromic congenital ichthyoses with widely varying severity. The group comprises lamellar ichthyosis, which is most often due to tranglutaminase−1 deficiency (Table 3), congenital ichthyosiform erythroderma, and the most severe but rare subtype of harlequin ichthyosis [12]. Newborns can be born with a tight and shiny stratum corneum, which is associated with ectropion, eclabium, fluid loss, and thermal dysregulation, and resulting in potentially life-threatening complications. However, the clinical presence of a collodion membrane is also encountered in other ichthyoses [5].

**Figure 3:**
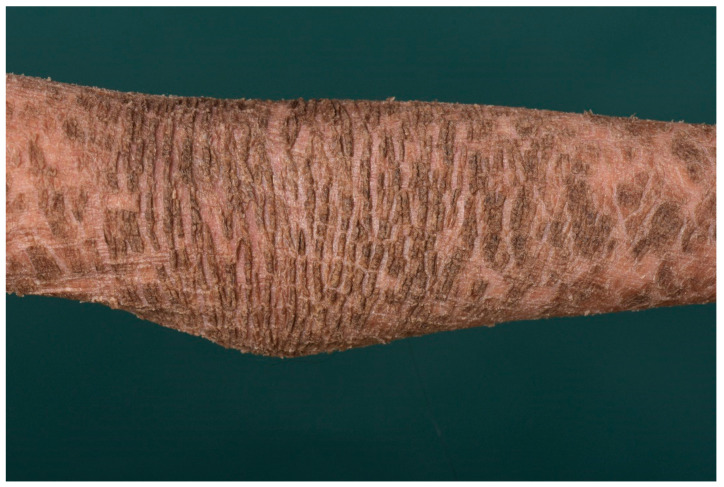
Lamellar ichthyosis. Dark brownish lamellar scaling in a patient with transglutaminase−1 deficiency.

Later, the collodion membrane is replaced by dark brown, adherent, plate-like scales (classic lamellar ichthyosis; Figure 3) or a whitish, poorly adherent, fine scale on reddened skin (non-bullous congenital ichthyosiform erythroderma). To varying degrees, there are associated palmoplantar keratoderma, nail dystrophies, fibrosing alopecia, and hypohidrosis with heat intolerance [5].

The ARCI forms are caused by different mutations. In 30–40% of cases, a mutation is present in the *transglutaminase−1* gene, resulting in a disruption of protein cross-linking and the esterification of ceramides in corneocytes. Using biotinylated donor substrates, such as the amine donor monodansylcadaverine, transglutaminase activity can be visualized immediately in situ by fluorescence labeling based on the incorporation of monodansylcadaverine on sections of unfixed frozen biopsies [13]. Mutations are also present in the *ATB-binding cassette transporter (ABCA12)* gene, which, unlike harlequin-ichthyosis, has residual activity in milder ARCI cases. ABCA12 is required in epidermal lipid transport via the lamellar bodies. Other mutations involve the *ichthyin, lipoxygenase, or cytochrome P450 oxidase* genes *FLJ39501*. A definitive correlation of this mutation with a specific phenotype of ARCI has not been fully established [8].

### 4.1. Histology

**Figure 4:**
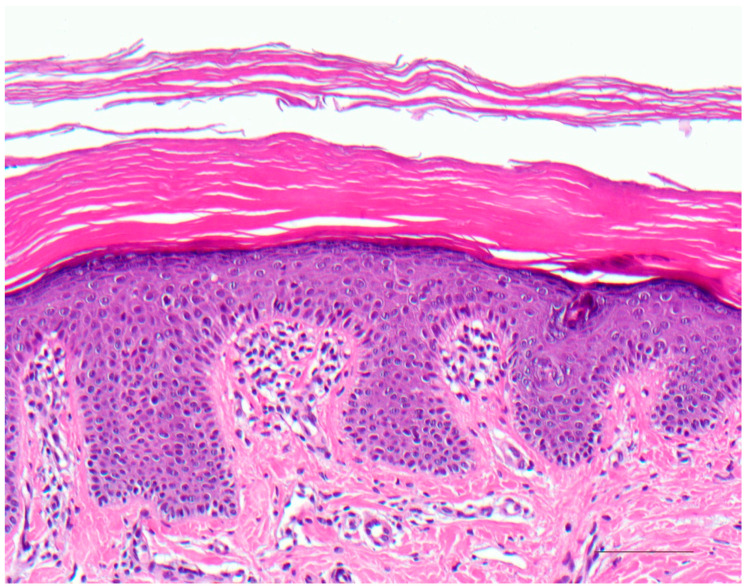
Autosomal recessive lamellar ichthyosis. Acanthotic epidermis with well-developed stratum granulosum and compact orthohyperkeratosis without further signs of inflammation. HE stain, original magnification, bar = 100 µm.

Histologically, there is compact orthohyperkeratosis, a slightly widened stratum granulosum, acanthosis, and papillomatosis of the epidermis. In the papillary body, the vessels appear dilated and spiraling, and lymphocytic infiltrates are scarce or mild (Figure 4) [7].

### 4.2. Differential Diagnoses

The various forms of ARCI cannot be differentiated histologically, except for harlequin ichthyosis. Similarly, X-linked ichthyosis presents with almost identical pathology. Lichen simplex chronicus presents with more severe inflammation and fibrosis in the papillary body.

## 5. Keratinopathic Ichthyosis

**Figure 5:**
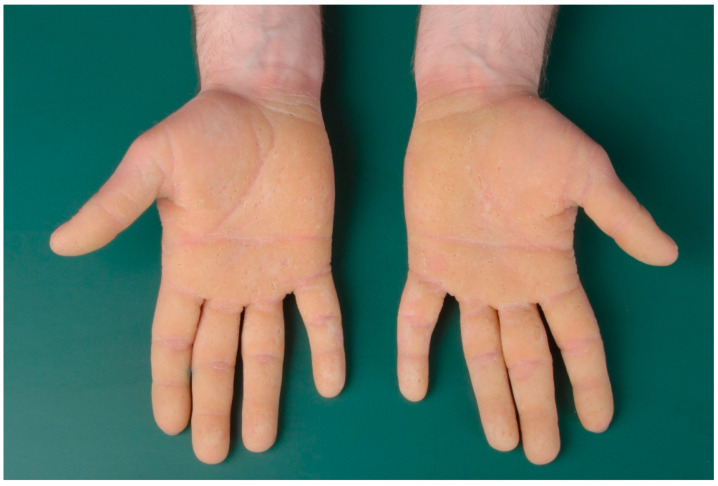
Epidermolytic ichthyosis. Diffuse palmoplantar keratoderma (*keratin 1* mutation).

Epidermolytic ichthyosis, formerly also called bullous congenital ichthyotic erythroderma Brocq, is due to a mutation of *keratin 1* or *keratin 10* and is therefore classified as keratinopathic ichthyosis (Table 3) [5]. Neonates present with erythroderma with blistering, sometimes pronounced, and later develop (spiky) keratoses, preferentially on the extremities (Figure 5). Patients with a *keratin 1* mutation also have palmoplantar keratosis, which is absent in patients with a *keratin 10* mutation because this keratin is not expressed there [14].

### 5.1. Histology

**Figure 6:**
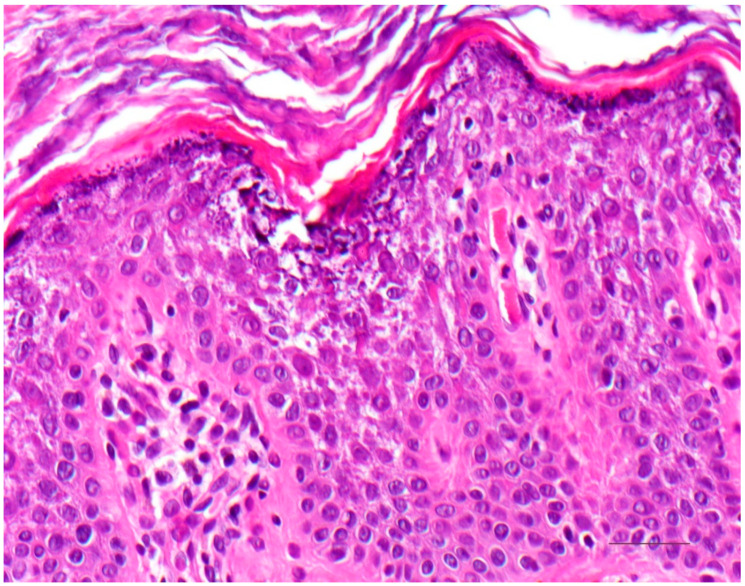
Epidermolytic ichthyosis. Acanthotic epidermis with massive orthohyperkeratosis. Suprabasal keratinocytes vacuolated with distinct hypereosinophilic granules and irregular keratohyalin granules. HE stain, bar = 50 µm.

There is massive orthohyperkeratosis and acanthosis of the epidermis. The suprabasal keratinocytes reveal vacuolization and distinct hypereosinophilic granules. In the stratum granulosum, the keratohyalin granules are coarse and irregular. The boundaries between keratinocytes are poorly demarcated, and clefts and blisters occur. Minor lymphocytic infiltrates may impose in the dermis (Figure 6) [8].

Electron microscopically, the hypereosinophilic granules correspond to clumps of the keratin skeleton. A collapse of the mutant keratins causes the vacuolar aspect of the cytoplasm and results in mechanical instability.

### 5.2. Differential Diagnoses

The histologic reaction pattern of epidermolytic hyperkeratosis is also found in superficial epidermolytic ichthyosis with a *keratin 2* mutation (ichthyosis bullosa Siemens), or epidermal nevi in the setting of mosaicism of keratinopathic ichthyoses [15,16]. Very discrete and circumscribed, these changes are also found incidentally in normal skin (preferentially in the vicinity of epithelial or melanocytic tumors), as well as in cysts, scars, or various inflammatory dermatoses.

## 6. Erythrokeratoderma

Erythrokeratodermas are defined by localized erythematous keratoses on the body and are now classified in the ichthyosis group [5]. They are caused by mutations of connexin 30.3 or 31 (Table 3). These transmembrane protein gap junctions are essential for intercellular communication and, thus, for epidermal differentiation [17].

Autosomal dominantly inherited erythrokeratodermia variabilis (Mendes da Costa syndrome) initially manifests with migratory figured erythema, and later persistent keratoses. The expression varies between intra- and interfamilial, and sometimes only circumscribed keratoses are found on pressure-exposed areas of the sole of the foot. Progressive symmetric erythrokeratodermia (Gottron) is no longer distinguished as a separate entity from erythrokeratodermia variabilis [18].

### Histology

**Figure 7:**
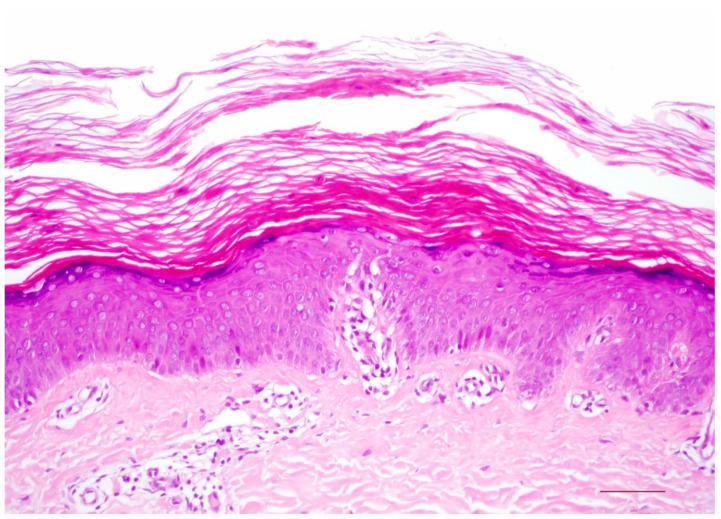
Erythrokeratoderma. Acanthotic epidermis with orthohyperkeratosis, focal parakeratosis, dyskeratotic keratinocytes, and preserved stratum granulosum. Discrete superficial perivascular lymphocytic infiltrate. HE stain, bar = 100 µm.

The epidermis shows acanthosis and undulating surface with hyperkeratosis, focal parakeratosis, dyskeratotic keratinocytes, and preserved stratum granulosum. Superficial perivascular lymphocytic infiltrate may be present (Figure 7). Overall, the histologic changes mentioned are highly variable and complicate diagnosis. The deficiency of the affected connexin can be easily visualized by immunohistochemistry; at the same time, compensatory connexin 43 expression is increased.

## 7. KID Syndrome and HID Syndrome

Keratitis–ichthyosis–deafness (KID) syndrome and hystrix-like–ichthyosis–deafness (HID) syndrome are different forms of an autosomal dominant inherited ichthyosis caused by a mutation of connexin 26 (Table 3) [19]. Because this connexin performs important functions in the inner ear, neurosensory hearing loss also exists. Patients with KID syndrome develop sharply circumscribed wart-like hyperkeratotic plaques on the face and extremities; in HID syndrome, hystrix-like generalized ichthyosis predominates. In the setting of this syndromic ichthyosis, keratitis, alopecia, nail dystrophy, dental abnormalities, or hypohidrosis, and an increased risk of infection and carcinoma occur.

### 7.1. Histology

**Figure 8:**
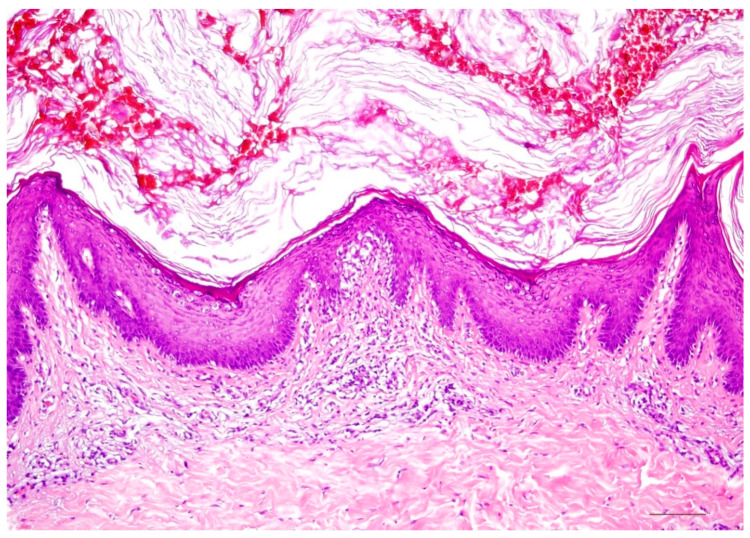
KID syndrome (keratitis–ichthyosis–deafness). Dyskeratotic keratinocytes with perinuclear halo (“bird’s eye”). HE stain, bar = 100 µm.

The epidermis is acanthotic with a partially verruciform appearance. The hyperkeratotic stratum corneum contains parakeratoses with large round nuclear remnants, and occasionally shadow cells with vacuolated nuclei (Figure 8). Dyskeratotic keratinocytes with perinuclear halo (“bird’s eye”) appear as a dominant criterion. The stratum granulosum may be absent or strongly pronounced. Subepidermal dense lymphocytic infiltrates occur in some cases. The openings of the hair follicles and sweat glands are highly keratinized. The sweat glands may be diminished and atrophic. Highly differentiated squamous cell carcinoma may also occur at a young age [8].

### 7.2. Differential Diagnoses

Verrucae vulgares also show vacuolated cells, but in KID/HID syndrome these persist in the stratum corneum. Vacuolization is absent in erythrokeratodermia.

## 8. Ichthyoses with Inflammatory Psoriasiform Pattern

Some hereditary ichthyoses have a histologic pattern that closely resembles psoriasis vulgaris or chronic dermatitis in the setting of atopic eczema, which is why misdiagnosis is common (Table 2). Because of the significant and sometimes lethal complications associated with this group of ichthyoses, prompt dermatohistologic diagnosis is important [20].

## 9. Netherton Syndrome

**Figure 9:**
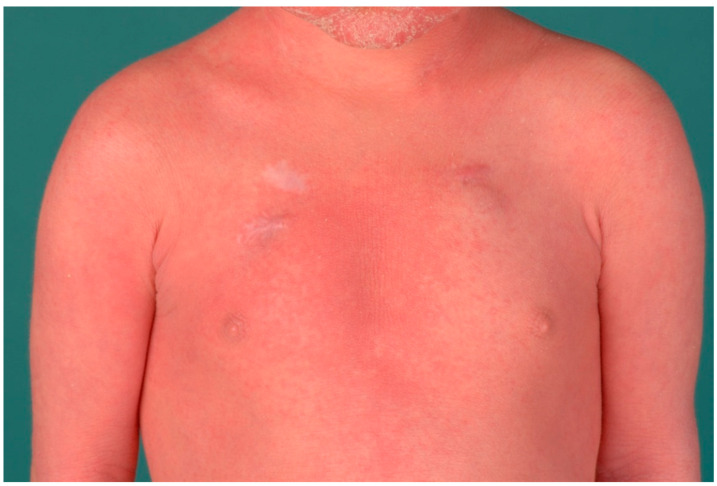
Netherton syndrome. Erythema and scaling of the trunk and face.

In autosomal recessive Netherton syndrome, there is a mutation of the *SPINK5* gene, which encodes LEKTI (“lymphoepithelial Kazal-type related inhibitor”), a major serine protease inhibitor of the epidermis and thymus (Table 3) [21]. Patients are born with marked erythroderma, which later often changes into anular eyrthema with a typical double-edged scale (“ichthyosis linearis circumflexa”) (Figure 9). Later, brittle hairs are also noticeable (“bamboo hairs”, trichorrhexis invaginata). Type 1 allergies, elevated IgE levels, and hypereosinophilia, as well as immunodeficiency and enteropathy, which can lead to massive failure to thrive, especially in the first year of life, are associated with this cornification disorder. Electrolyte disturbances and sepsis are lethal risks for infants.

### 9.1. Histology

**Figure 10:**
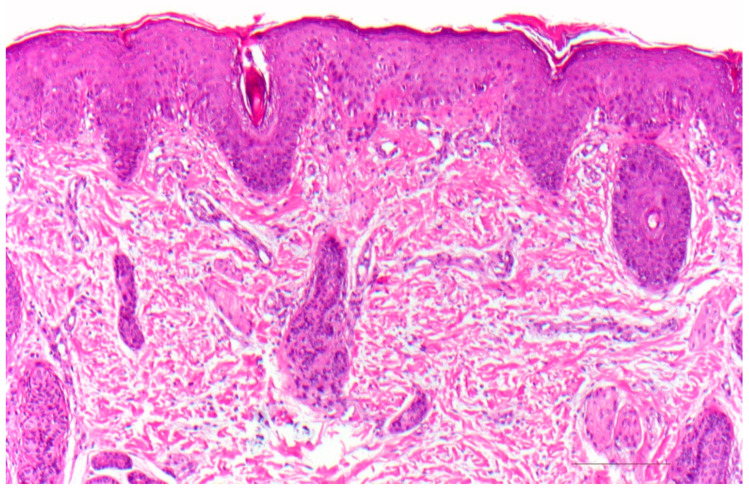
Netherton syndrome. Regular (psoriasiform) hyperplasia with focal parakeratosis and thinned stratum granulosum. Dilated vessels in the papillary dermis and inflammatory infiltrates. HE stain, bar = 100 µm.

**Figure 11:**
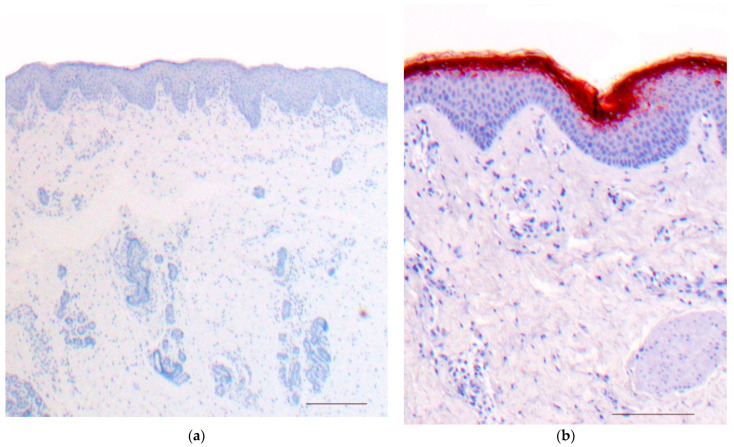
Netherton syndrome, immunohistochemistry, bar = 200 µm (**a**,**b**). Immunohistochemistry shows a lack of staining for LEKTI in the epidermis and hair follicles (**a**); regular expression of LEKTI in the upper layers of the epidermis of healthy skin, bar = 200 µm (**b**). Immunoperoxidase staining.

There is psoriasiform hyperplasia with a moderately widened stratum corneum showing focal parakeratosis and accumulations of neutrophils. The stratum granulosum is absent or severely diminished. The papillary dermis is papillomatously elongated and contains dilated vessels and inflammatory infiltrates with lymphocytes, neutrophils, and eosinophilic granulocytes (Figure 10). Sometimes, however, there are histologic changes, as found in atopic dermatitis. Immunohistochemically, staining for LEKTI is absent in the epidermis and hair follicles (Figure 11) [22].

### 9.2. Differential Diagnoses

Psoriasis vulgaris or atopic dermatitis cannot always be differentiated histologically. PAS-positive granules in the stratum corneum cannot always be detected and are not specific. The immunohistochemically detectable lack of LEKTI expression is important evidence for Netherton syndrome. Other forms of ichthyosis with psoriasis-like histology are listed in Table 2.

## 10. Peeling Skin Disease

**Figure 12:**
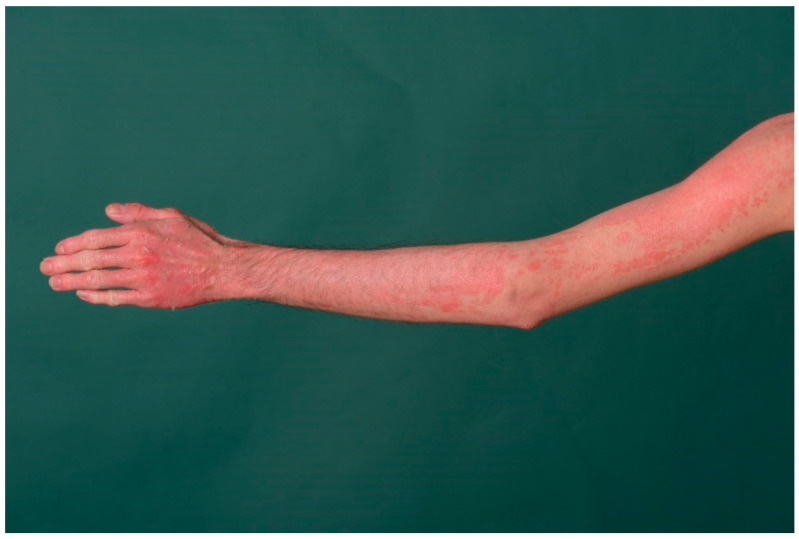
Peeling skin disease. Diffuse erythema with superficial skin detachment is evident from birth and persists throughout life with seasonal variation.

In peeling skin disease (peeling skin syndrome B), generalized erythema with superficial skin detachment is evident from birth and persists throughout life with seasonal variation (Figure 12). In addition, episodic detachment of the nail plates (onychomadesis) may occur. Hair status is inconspicuous except for a transient slight epilation of fine hairs [23].

There is a mutation of corneodesmosin, an important adhesion protein expressed in the extracellular sections of desmosomes in the stratum corneum of the epidermis, as well as at the inner hair root sheath of hair follicles (Table 3). Ultrastructurally, there is detachment of intact corneocytes from the stratum granulosum (extracellular cleft formation) [20,23]. Autosomal dominant mutations in other domains of corneodesmosin cause hypotrichosis simplex.

Concomitant barrier disruption leads to inflammation with massive pruritus, urticaria, angioedema, food allergy, and asthma with elevated IgE levels and blood eosinophilia.

### 10.1. Histology

The epidermis is hyperplastic with prominent rete ridges. There is mild hyperkeratosis with focal parakeratosis and thinned stratum granulosum. Some biopsies show a focal detachment of the stratum corneum, and in some cases, the stratum corneum is completely absent. However, these changes cannot always be detected on a paraffin section. There are superficial and perivascular lymphocytic infiltrates with single neutrophils, which are also found in the stratum corneum. The papillary body is elongated and edematous, vessels are not dilated [7]. Immunohistochemically, staining for corneodesmosin is absent in the stratum corneum [23].

### 10.2. Differential Diagnosis

Psoriasis vulgaris, Netherton syndrome, and CHILD syndrome cannot be differentiated without immunohistochemistry.

## 11. CHILD Syndrome

Congenital Hemidysplasia with Ichthyosiform nevus and Limb Defect (CHILD) syndrome is a very rare X-linked dominant disorder that is usually lethal for male offspring. It is characterized by unilateral inflammatory, often waxy, yellow skin lesions, emphasized in the large flexures and perianogenital region [8,24]. Extracutaneous symptoms range from discrete hypoplasia of the limbs to severe deformities. Monosymptomatic cases are often misdiagnosed as psoriasis or ILVEN [25].

The disorder is caused by nonsense or missense mutations in the so-called *NSDHL* gene, which lead to a disturbance of cholesterol biosynthesis (Table 3) [26,27].

### 11.1. Histology

Hyperplastic epidermis with elongated rete ridges and marked orthohyperkeratosis with focal parakeratosis are found. The stratum granulosum may be prominent in some areas but can also be absent. A perivascular lymphocytic infiltrate and xanthomatous macrophages that are markedly immunoreactive for adipophilin are apparent in the papillary body [7].

### 11.2. Differential Diagnoses

Verruciform xanthomas also contain xanthomatous macrophages, which are absent in the other major differential diagnoses as psoriasis inversa and epidermal nevus. The presence of verruciform xanthomas or verruciform xanthoma-like changes in the setting of CHILD syndrome is possible.

## 12. Severe Dermatitis, Multiple Allergies, Metabolic Wasting Syndrome (SAM Syndrome)

SAM syndrome was identified as a severe life-threatening genodermatosis by Liat Samuelov et al. in 2013 [28]. The acronym stands for severe dermatitis, multiple allergies, metabolic wasting syndrome. It is caused by a mutation of desmoglein−1 (DSG1) (Table 3), a major desmosomal adhesion molecule also involved in pemphigus disease. Later, a *desmoplakin* mutation was also identified [29]. The disease is inherited in an autosomal recessive manner; heterozygous carriers of the *DSG1* mutation develop only striate palmoplantar keratoderma [30].

Clinically, there is ichthyosiform erythroderma in newborns similar to autosomal recessive lamellar ichthyosis, Netherton syndrome, or peeling skin disease. Other symptoms are pruritus, hypotrichosis, food allergies with elevated IgE, dysphagia, decreased growth, and recurrent skin and respiratory infections. In varying degrees, pustular formation, palmoplantar keratoses, onychodystrophy, dental anomalies, cardiac abnormalities, and eosinophilic esophagitis are found. There is marked inter- and intrafamilial variability [30]. The accompanying inflammation can be explained by proinflammatory activity in keratinocytes in the context of impaired barrier function and downregulated blockage of signal transduction pathways [31]. Furthermore, the intracytoplasmic portion of DSG1 blocks the RAS-RAF signaling pathway and, thus, affects epidermal differentiation [32,33]. Similar to Netherton syndrome, anti-inflammatory therapies with biologics improve the clinical picture (Oji V, unpublished).

### 12.1. Histology

**Figure 13:**
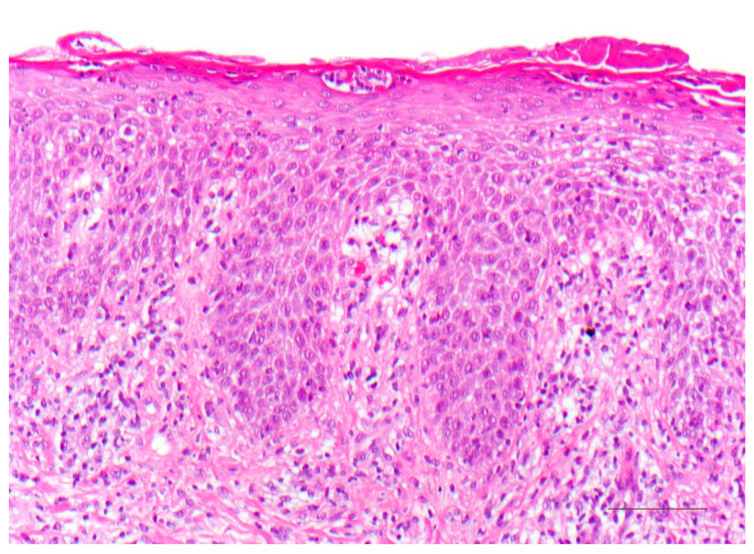
SAM syndrome. Psoriasiform dermatitis with dilated intercellular spaces of the epidermis without blistering (“desmosomal acantholysis”). HE stain, bar = 100 µm.

Histologically, there is a superficial lymphocytic dermatitis with hyperplastic epidermis, parakeratosis, and neutrophil granulocytes that strongly resembles psoriasis (psoriasiform dermatitis). However, there are typically dilated intercellular spaces of the epidermis without blistering (Figure 13). Intracellular edema and serum exudate (as in spongiotic dermatitis), and rounding of keratinocytes and pyknosis of nuclei (as in pemphigus disease), hypereosinophilia of cytoplasm (as in M. Darier or Hailey–Hailey), ballooning, and typical viropathic nuclear changes, as in herpes disease, are absent.

### 12.2. Differential Diagnoses

All genodermatoses with mutations of desmosomal proteins leading to the pattern of “desmosomal acantholysis” (Metze, unpublished). These include keratosis palmoplantaris areata et striata (striate palmoplantar keratoderma types 1 and 2), Carvajal-Huerta syndrome, Naxos syndrome, ectodermal dysplasia skin fragility syndrome (McGrath syndrome), or peeling skin disease [34].

## Figures and Tables

**Table 1:** Clinical classification of ichthyoses.

**Vulgar ichthyosis, isolated**
Ichthyosis vulgaris
X-linked recessive ichthyosis
**Vulgar ichthyosis, syndromic**
Refsum syndrome
Multiple sulfatase deficiency
**Congenital ichthyosis, isolated**
Keratinopathic ichthyosis
Autosomal recessive congenital ichthyosis (ARCI)
Harlequin ichthyosis (subtype of ARCI)
Autosomal dominant lamellar ichthyosis
Congenital reticular ichthyosiform erythroderma (CRIE, Confetti ichthyosis)
Ichthyosis hystrix type Curth–Macklin
Peeling skin disease
Erythrokeratodermia
and others
**Congenital ichthyosis, syndromic**
HID/KID syndrome
Netherton syndrome
CHILD syndrome
SAM syndrome
Conradi–Hünermann–Happle syndrome
Sjögren–Larsson syndrome
Chanarin–Dorfmann syndrome
Trichothiodystrophy
IFAP syndrome
and others

**Table 2:** Ichthyoses with a psoriasis-like picture.

Ichthyoses with Psoriasis-Like Picture
Netherton syndrome
Peeling skin disease
CHILD syndrome
Severe dermatitis, multiple allergies, metabolic wasting syndrome (SAM syndrome)
Anular epidermolytic ichthyosis

**Table 3:** Different types of ichthyosis with gene mutation and mode of inheritance.

**Non-Syndromic Ichthyoses**	**Common Ichthyoses**	Ichthyosis		*Gene* (mode of inheritance)
		Ichthyosis Vulgaris		*FLG (filaggrin)* (autosomal semidominant)
		X-Linked Ichthyosis		*STS (steroid sulfatase)* (X-linked recessive)
	**ARCI and Keratinopathic Ichthyoses**	Harlequin Ichthyosis		*ABCA12 (ATP Binding Cassette Subfamily A Member 12)* (autosomal recessive)
		Lamellar Ichthyosis, Congenital Ichthyosiform Erythroderma		*TGM1 (transglutaminase−1);* *ALOX12B (Arachidonate 12-Lipoxygenase, 12R Type); ALOXE3 (Arachidonate Lipoxygenase 3); CYP4F22 (Cytochrome P450 Family 4 Subfamily F Member 22); NIPAL4 (Ichthyin)* and others (autosomal recessive)
		Bathing Suit Ichthyosis		*TGM1* (autosomal recessive)
		Keratinopathic Ichthyoses	EI	*KRT1 (keratin 1*); *KRT10 (keratin 10)* (autosomal dominant, sometimes recessive (*KRT10* mutations)
			SEI	*KRT2 (keratin 2)* (autosomal dominant)
		Rare Variants of KPI	CRIE	*KRT1* *KRT10* (autosomal dominant, de novo mutations)
**Further Non-Syndromic Ichthyoses**		Peeling Skin Disease		*CDSN (corneodesmosin)* (autosomal recessive)
		Erythrokeratoderma Variabilis		*GJB3* (encoding Connexin 31) *GJB4* (encoding Connexin 30.3) (often autosomal dominant)
**Syndromic Ichthyoses**		Netherton Syndrome		*SPINK5* (encoding LEKTI) (autosomal recessive)
		KID Syndrome		*GJB2* (encoding Connexin 26) (autosomal dominant)
		CHILD Syndrome		*NSDHL* (*NAD(P) Dependent Steroid Dehydrogenase-Like*) (x-linked dominant)
		SAM Syndrome		*DSG1 (desmoglein−1)* *DSP (desmoplakin)* (autosomal recessive)

## Abbreviations

CHILD syndrome	Congenital hemidysplasia with ichthyosiform erythroderma and limb defects syndrome
CRIE	Congenital reticular ichthyosiform erythroderma
EI	Epidermolytic ichthyosis
KID syndrome	Keratitis ichthyosis deafness syndrome
SAM syndrome	Congenital erythroderma–hypotrichosis–recurrent infections–multiple food allergies syndrome
SEI	Superficial epidermolytic ichthyosis
